# The highly expressed 5’isomiR of hsa-miR-140-3p contributes to the tumor-suppressive effects of miR-140 by reducing breast cancer proliferation and migration

**DOI:** 10.1186/s12864-016-2869-x

**Published:** 2016-08-08

**Authors:** Omar Salem, Nese Erdem, Janine Jung, Ewald Münstermann, Angelika Wörner, Heike Wilhelm, Stefan Wiemann, Cindy Körner

**Affiliations:** Division of Molecular Genome Analysis, German Cancer Research Center (DKFZ), INF580, Heidelberg, 69120 Germany

**Keywords:** Breast cancer, IsomiRs, MiRNA, Seed sequence, Proliferation, Migration, MiR-140

## Abstract

**Background:**

miRNAs are small noncoding RNA molecules that play an important role in post-transcriptional regulation of gene expression. Length and/or sequence variants of the same miRNA are termed isomiRs. While most isomiRs are functionally redundant compared to their canonical counterparts, the so-called 5’isomiRs exhibit a shifted 5’ end and therefore a shifted seed sequence resulting in a different target spectrum. However, not much is known about the functional relevance of these isoforms.

**Results:**

Analysis of miRNA-seq data from breast cancer cell lines identified six pairs of highly expressed miRNAs and associated 5’isomiRs. Among them, hsa-miR-140-3p was of particular interest because its 5’isomiR showed higher expression compared to the canonical miRNA annotated in miRbase. This miRNA has previously been shown to control stemness of breast cancer cells. miRNAseq data of breast cancer patients (TCGA dataset) showed that both the canonical hsa-miR-140-3p and its 5’isomiR-140-3p were highly expressed in patients’ tumors compared to normal breast tissue. In the current work, we present the functional characterization of 5’isomiR-140-3p and the cellular phenotypes associated with its overexpression in MCF10A, MDA-MB-468 and MDA-MB-231 cell lines in comparison to the canonical hsa-miR-140-3p. Contrary to the effect of the canonical hsa-miR-140-3p, overexpression of the 5’isomiR-140-3p led to a decrease in cell viability. The latter observation was supported by cell cycle analysis, where the 5’isomiR-140-3p but not the hsa-miR-140-3p caused cell cycle arrest in G_0_/G_1_-phase. Additionally, 5’ismoiR-140-3p overexpression was found to cause a decrease in cell migration in the three cell lines. We identified three novel direct target genes of the 5’isomiR-140-3p; *COL4A1*, *ITGA6* and *MARCKSL1*. Finally, we have shown that knocking down these genes partially phenocopied the effects of the 5’isomiR-140-4p overexpression, where *COL4A1* and *ITGA6* knockdown led to reduced cell viability and cell cycle arrest, while *MARCKSL1* knockdown resulted in a decrease in the migratory potential of cells.

**Conclusions:**

In summary, this work presents evidence that there is functional synergy between the canonical hsa-miR-140-3p and the newly identified 5’isomiR-140-3p in suppressing growth and progression of breast cancer by simultaneously targeting genes related to differentiation, proliferation, and migration.

**Electronic supplementary material:**

The online version of this article (doi:10.1186/s12864-016-2869-x) contains supplementary material, which is available to authorized users.

## Background

Breast cancer is a complex heterogeneous form of cancer with lots of genetic alterations. According to the American Cancer Society, excluding the cancers of skin, breast cancer is the most common cancer among women, accounting for nearly one in three cancers diagnosed in US women [1]. In breast cancer, it is not the primary tumor, but its metastases at different distant sites that are the main cause of death [2]. Based on different phenotypes and gene expression profiling, breast cancer can be divided into five major so-called PAM50 subtypes: luminal A, luminal B, tumor enriched with human epidermal growth factor receptor 2 (Her2), basal-like and normal-like subtype [3–6]. Luminal subtypes are characterized by the expression of estrogen receptor (ER) and are treated with adjuvant endocrine therapy targeting the ER signaling such as tamoxifen and aromatase inhibitors [6]. The third subtype, Her2 positive, is classified by the absence of ER and progesterone receptor (PR) expression, high expression of Her2 and high proliferation rate [6]. Her2 positive tumors can be targeted using an antibody against Her2 such as trastuzumab [7, 8]. Basal-like breast cancer generally expresses none of the three markers (ER, PR and Her2), and largely corresponds to the group of triple negative breast cancer (TNBC). It is often highly aggressive and is associated with poor prognosis [9]. No targeted therapies are currently available for this breast cancer subtype and current regimes include the conventional chemotherapeutic approach. Nevertheless, promising strategies are being developed to treat TNBC, such as poly-ADP ribose polymerase-1 inhibitors [6, 10, 11]. Normal-like subtype is the rarest form of breast cancer. It is relatively poorly characterized, yet has been shown to express ER, Her2 and PR. Its clinical prognosis is believed to lie between TNBC and luminal A subtypes [6].

MicroRNAs (miRNAs) are single stranded RNA molecules of ~22 nucleotides that are associated with the Argonaute proteins (AGO). They serve a role in post-transcriptional gene regulation in both plants and animals. miRNAs are generated by the sequential action of two RNase III-type proteins (Drosha and Dicer) on short hairpin RNAs. Upon cleavage by Dicer, the small RNA duplex is incorporated into a complex known as RNA-induced silencing complex (RISC) or in case of miRNAs referred to as miRISC [12–14].

miRNAs control most of the protein-coding genes resulting in control of cancer-relevant processes such as cell cycle, proliferation, differentiation, apoptosis, and migration. miRNAs associated with cancers are termed oncomiRs. A single miRNA can target hundreds of mRNAs. Hence, even though the regulation of gene expression by miRNAs is generally rather mild, aberrant expression of a single miRNA may affect a multitude of transcripts involved in cancer-related signaling pathways. This fact makes the situation more complex, as the overall functions of miRNAs in oncogenesis can be context dependent. Accordingly, a particular miRNA may be found to be upregulated in some cancers while being downregulated in others [15]. These findings became more pronounced with the availability of whole genome mRNA and miRNA expression data from different cancers [16–20]. In addition, mouse models with miRNA overexpression or ablation have shown a direct causal link between miRNA expression profiles and cancer development. Clinically, miRNA are studied as clinical biomarkers and/or putative therapeutic targets [21, 22].

Deep sequencing technologies have enabled the more extensive study of miRNAs, leading to the identification of different variants of the same miRNA. Typically, miRNAs are annotated as a single defined sequence and despite that, for many miRNAs, length and/or sequence variants have been observed, these variants were believed to be experimental artefacts and were thus ignored or dismissed [23]. However, massive parallel sequencing and computational algorithms have confirmed the biological existence of such variants in different species [24, 25]. The term isomiR was coined to describe the different sequence and/or length variants of an individual miRNA. Additionally, studies demonstrated the active associations between isomiRs and RISC, suggesting that they can interact with mRNAs [26]. These observations pointed to the possible physiological role of isomiRs. Different reports pointed to gender and/or race dependence of the isomiRs expression [27, 28]. However, the regulatory mechanisms underlying the production of specific isomiRs are not fully understood. Moreover, to date, there have been only few reports on the functional role of lowly abundant 5’isomiRs and the capacity to modulate their targets relative to the canonical counterparts [28, 29]. However, the functional importance of highly expressed 5’isomiRs has not been addressed, yet.

Generally, isomiRs can broadly be classified into three main categories: 5’ isomiRs, 3’ isomiRs and polymorphic isomiRs. 3’ and 5’ isomiRs are those having variations at the 3’ and 5’ ends of the mature miRNA sequence respectively, whereas polymorphic isomiRs are harboring different internal nucleotide sequences [23]. Different mechanisms contribute to the generation of isomiRs. In the canonical miRNA biogenesis pathway 5’ and 3’ ends are specified by consecutive cleavage events of the primary transcript by the ribonucleases Drosha and Dicer. The alternative cleavage by Drosha and/or Dicer could result in length variation which has been shown to partially depend on individual features such as ethnical background [27, 28, 30]. Furthermore, the 3’ ends of the RISC-bound miRNAs are liable to trimming by exoribonucleases [31, 32]. In the latter two cases the product is termed “templated”, since the miRNA sequence still matches the parent gene. Post-transcriptional addition of one or more bases might also take place with the help of nucleotidyltransferases. Most of the nucleotidyltransferases are 5’- 3’ polymerases leading to the formation of 3’ isomiRs [33]. The addition of nucleotides that do not match the parent gene is termed as “non-templated” addition [34]. RNA editing, especially adenosine to inosine editing (A-to-I) is suggested to be a major driver in the generation of polymorphic isomiRs [35]. Most miRNAs, however, do not exhibit a high frequency of editing. The most frequently observed type of isomiR in animals and plants is the 3’ isomiR. 5’ and polymorphic isomiRs are generally rare. Nevertheless, they still represent a significant proportion of the population of some miRNAs [36, 37]. 3’ isomiRs or non-seed-based nucleotide substitutions are believed to be functionally redundant and mainly affect miRNA stability. In contrast, it has been shown that 5’ isomiRs can have different mRNA targets and thereby different phenotypes compared to their canonical counterparts [27, 28, 34].

MiR-140 is encoded within intron 16 of *Wwp2*, an E3 ubiquitin ligase. It was first identified as a key player in cartilage development and homeostasis in chondrocytes [38]. The regulation of miR-140 was reported to be tissue-dependent. Besides its role in chondrocytes, it was found to be expressed in numerous other tissues and cell types including brain, breast, lung, colon, ovary and testis [39–41]. Importantly, expression-profiling experiments revealed a potential tumor suppressor function for miR-140 in many cancers where its expression was found to be downregulated in cutaneous squamous cell carcinoma, basal cell carcinoma, osteosarcoma, ovarian cancer, colon cancer and lung squamous cell carcinoma. Inversely, Zou et al. revealed a correlation between miR-140-3p overexpression with chordoma invasion and recurrence suggesting poor prognosis [42].

In the majority of miRNA species, the 5’ miRNA is annotated as the guide strand, while the complimentary 3’ miRNA is degraded. Interestingly enough, stably expressed levels of miR-140-3p as well as miR-140-5p were found to be present. The expression was found to be tissue dependent and each strand had its own function owing to different seed sequences [43]. Wolfson et al. reported a tumor suppressor role for miR-140 mediated by Wnt, SOX2 and SOX9 stem cell regulator pathways [44]. Downregulation of miR-140 in breast cancer was attributed to methylation of CpG islands in the promoter region of the miRNA gene. Inhibition of miR-140 allows for uncontrolled elevation of SOX2, which is known to be a stem cell self-renewal regulator causing an increase in stem cell populations and breast cancer progression, initiation and growth [44]. miR-140-3p is one of few miRNAs that showed high expression of 5’isomiR according to miRBase [45].

In the present work, we sought to characterize the effects of the overexpression of both the canonical hsa-miR-140-3p and the 5’isomiR-140-3p on breast cell lines through functional assays that assess cell viability, cell cycle, apoptosis and migration. Moreover, we aimed at identifying and validating target genes for 5’isomiR-140-3p that could account for the observed phenotypes. In summary, we could attribute tumor-suppressive phenotypes including reduced proliferation due to cell cycle arrest in G_0_/G_1_ phase of the cell cycle as well as reduced cell migration specifically to the 5’isomiR-140-3p. These phenotypes could be linked to direct targeting of *MARCKSL1*, *ITGA6* and *COL4A1*. Analysis of two major breast cancer patient cohorts (METABRIC and TCGA) confirmed the relevance of both miRNA and the novel target genes, especially in TNBC.

## Results

### 5’isomiR-140-3p is highly expressed in breast cancer cell lines and patients

With the advent of miRNA sequencing, the existence of isomiRs became increasingly clear. In addition, it is now possible to quantitatively assess expression of miRNAs and their isomiRs to allow for estimations of their biological relevance. Combining these two means of information, we analyzed miRNA sequencing datasets of several breast cancer cell lines that had been generated as part of the Illumina iDEA challenge to identify pairs of highly expressed miRNAs and 5’isomiRs. Here, we neglected variants at the 3’end of both species, treating all 3’isomiRs with a length of 18–24 nucleotides as miRNA or 5’isomiR, respectively, depending on their 5’ end. Using the notation suggested by Loher and colleagues [27], we refer for example to all miRNA species between hsa-miR-140-3p 0|-3 and 0|+3 (in Table 1 simplified as 0|x) as canonical hsa-miR-140-3p, whereas all species between hsa-miR-140-3p +1|-2 to +1|+3 (in Table 1 simplified as +1|x) are considered as 5’isomiR-140-3p. The sequencing data is provided as Additional file 1 and the raw data as well as more detailed information about the experimental setup can be found at ArrayExpress (E-MTAB-4539).

**Table 1:** Expression (in rpm) of pairs of highly expressed miRNAs/5’isomiRs in a panel of breast cancer cell lines

Notation of isoform		Seed sequence	MCF7	MDA-MB-231	T47D	BT20	BT474	MDA-MB-468	ZR-75	MCF10A
miR-10a-5p 0|x	canonical	ACCCTGT	81.9	4923.9	9254.5	7698.9	96.5	181.4	854.1	582.7
miR-10a-5p +1|x	isomiR	CCCTGTA	56.9	2158.4	3911.3	3660.0	64.2	65.0	477.0	273.0
miR-1307-3p 0|x	canonical	CTCGGCG	1159.2	737.2	2299.7	575.6	1894.2	554.8	2560.7	290.0
miR-1307-3p +1|x	isomiR	TCGGCGT	394.2	253.2	943.8	222.5	643.7	348.3	784.3	72.7
miR-140-3p 0|x	canonical	ACCACAG	180.9	486.8	117.4	289.0	149.1	63.0	181.2	199.2
miR-140-3p +1|x	isomiR	CCACAGG	407.7	851.6	284.8	568.7	217.6	95.2	306.2	332.8
miR-183-5p 0|x	canonical	ATGGCAC	1187.2	91.7	1065.9	1132.5	2017.2	1028.1	849.9	518.7
miR-183-5p +1|x	isomiR	TGGCACT	1102.0	44.0	831.3	822.5	1860.5	767.9	725.6	312.4
miR-203-3p 0|x	canonical	TGAAATG	2327.2	3.1	2514.6	544.1	5319.0	427.8	2870.3	71.3
miR-203-3p +1|x	isomiR	GAAATGT	2115.5	1.5	1137.8	329.8	2116.1	175.1	1104.8	23.1
miR-30a-3p 0|x	canonical	TTTCAGT	13.9	991.7	162.5	191.8	30.8	1033.2	108.8	239.7
miR-30a-3p +1|x	isomiR	TTCAGTC	3.7	305.1	53.5	65.8	6.6	311.7	35.8	44.1

As criteria, we defined an expression of the canonical miRNA of at least 100 rpm on average and a ratio between 5’isomiR and miRNA of at least 1 to 5 on average. Thereby, we identified six potentially interesting miRNA/5’isomiR pairs (Table 1). Interestingly, we found that in case of one of these miRNAs, namely miR-140-3p, expression of the isomiR was even higher compared to the canonical form. Therefore, we decided to focus on this pair.

We analyzed the breast cancer patients’ sequencing data from TCGA for the expression of the canonical hsa-miR-140-3p and its 5’isomiR-140-3p [46]. The clinical characteristics as well as the expression of canonical miRNA and 5’isomiR are listed in Additional file 2. The abundance of the different 3’isomiRs of the two 5’isomiRs under investigation is detailed in Additional file 3 listing the average expression of each miRNA species in the analyzed patient samples.

**Fig. 1:**
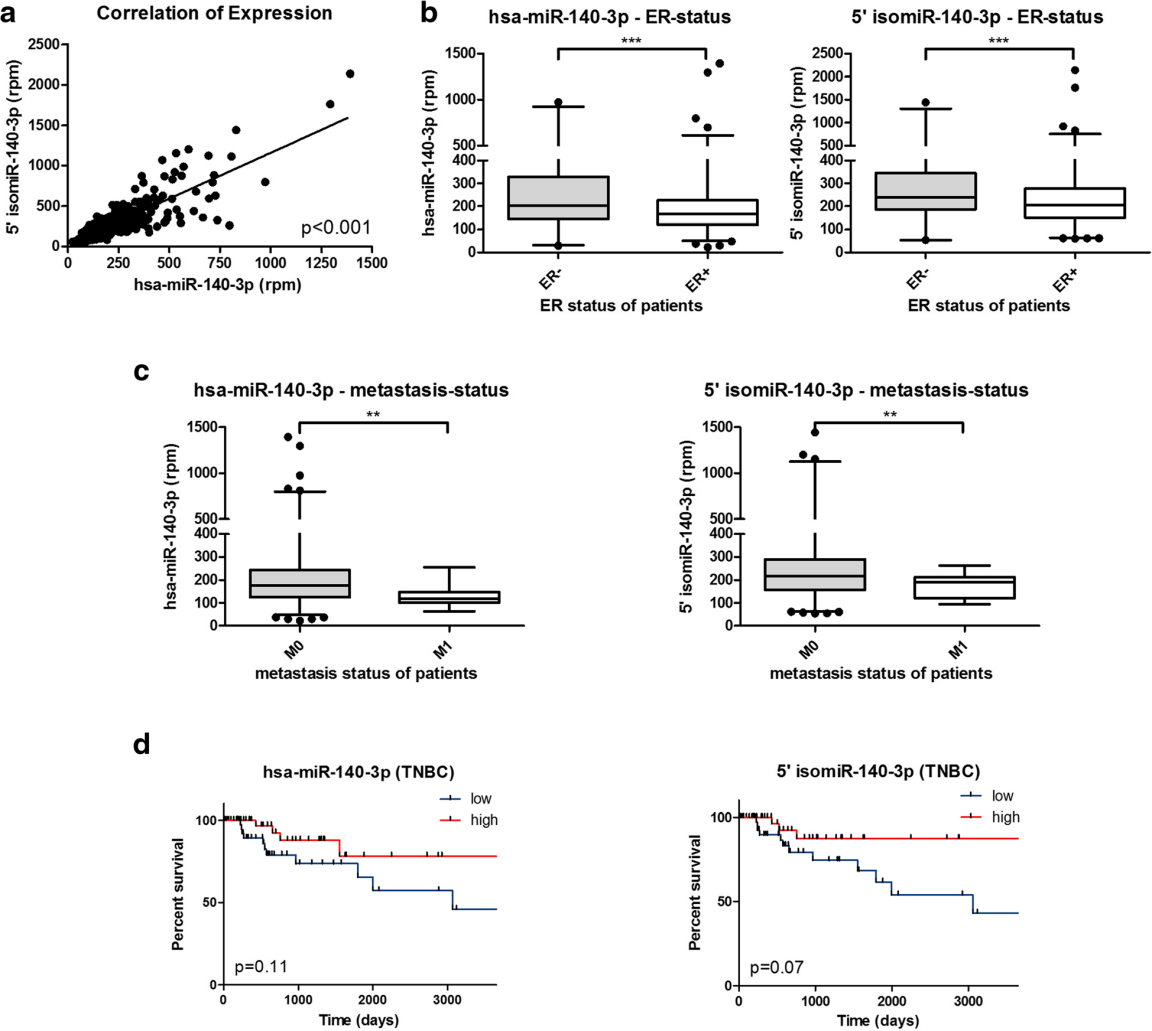
Statistical analysis of Breast Cancer patient data from TCGA [46]. Sequencing data from 616 patients were analyzed for miRNA expression levels of the canonical hsa-miR-140-3p and the 5’ isomiR-140-3p. **a** Expression of the hsa-miR-140-3p and 5’isomiR-140-3p show strong positive correlation. **b** hsa-miR-140-3p and 5’isomiR-140-3p levels are higher in ER- patients. **c** Distant metastases correlate with lower expression of both hsa-miR-140-3p and 5’ isomiR-140-3p. **d** TNBC patients with high expression levels of hsa-miR-140-3p and 5’ isomiR-140-3p show a trend of better survival

Both isoforms were found to be strongly positively correlated (*p* < 0.001, *r* = 0.85; Fig. 1a). The patients from the same dataset were grouped according to ER expression and the levels of hsa-miR-140-3p and 5’isomiR-140-3p were analyzed. It was found that both the hsa-miR-140-3p and 5’isomiR-140-3p were higher in ER negative (ER-) patients (Fig. 1b). Furthermore, patients with distant metastases had lower expression of both isoforms compared to patients without metastases (Fig. 1c). Additionally, survival analysis in TNBC patients showed a trend of better survival among patients with higher expression levels of both hsa-miR-140-3p and 5’isomiR-140-3p (Fig. 1d); a trend that was not observed when analyzing the overall population of breast cancer patients (data not shown).

### 5’isomiR specifically inhibits proliferation and migration of breast cell lines

**Fig. 2:**
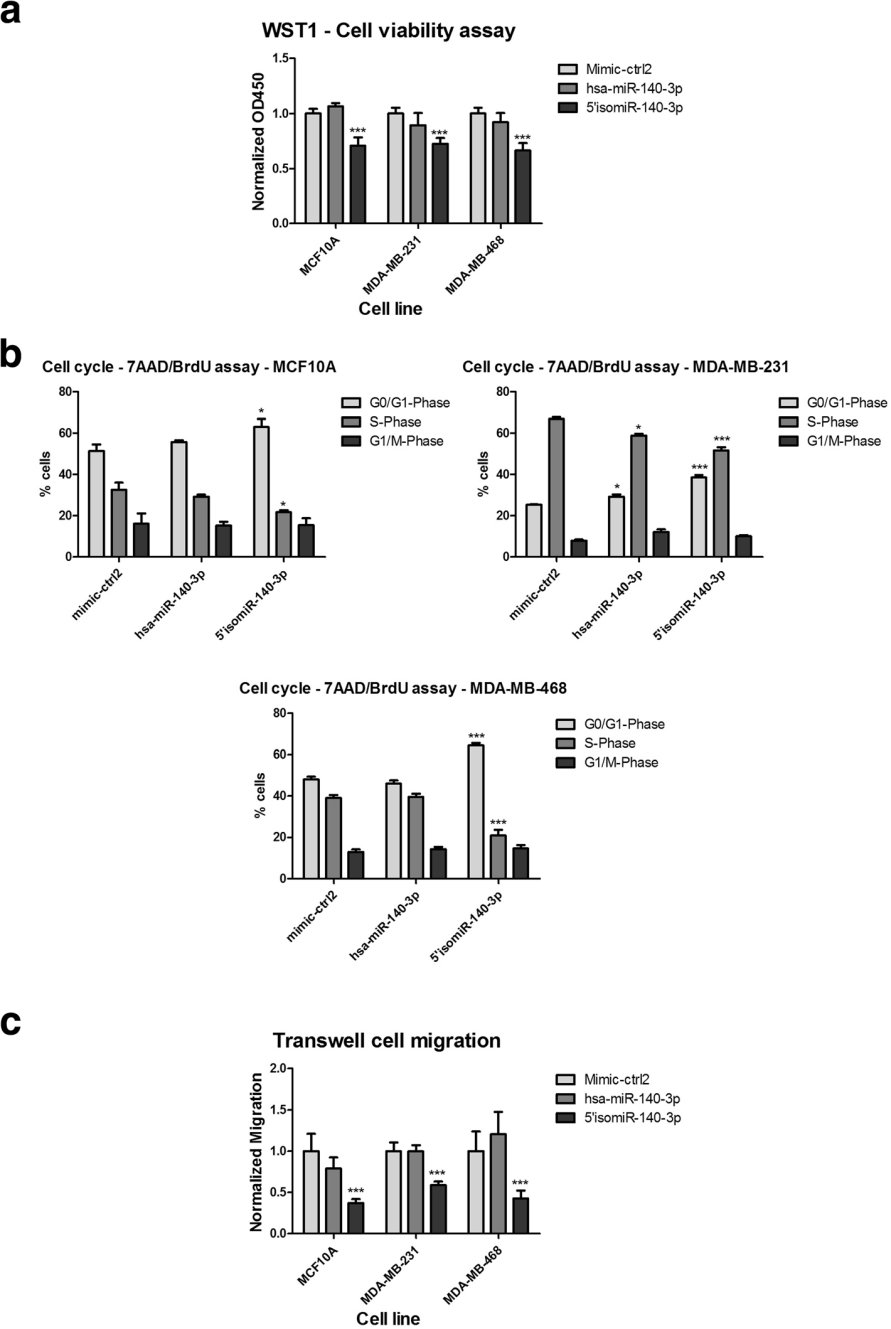
Effect of 5’ isomiR-140-3p overexpression on cell viability, cell cycle and cell migration. **a** and **b** MCF10A, MDA-MB-231 and MDA-MB-468 cells were transfected with miRNA mimics (hsa-miR-140-3p and 5’isomiR-140-3p), or mimic-ctrl2 (as negative control), then were incubated for 72 h. **a** WST-1 reagent was added to the cells and absorbance at 450 nm was measured. Results were normalized to the negative control. Data are presented as average of 6 biological replicates ± standard deviation indicated as error bars. **b** MCF10A, MDA-MB-231 and MDA-MB-468 cells were transfected with miRNA mimics, 24 h after transfection starved in serum-free media for 24 h and allowed to re-enter the cell cycle for 24 h in full growth medium. Cells were incubated with BrdU for 30 min before fixation and lysis and stained with anti-BrdU-FITC and 7-AAD for subsequent FACS analysis. Data are presented as average and standard deviation of three biological replicates. **c** Cells were transfected in 6-well plates with miRNA mimics (hsa-miR-140-p3 and 5’ isomiR-140-3p) or mimic miRNA negative control. 48 h later, cells were reseeded in transwell inserts (with 8.0 μm polycarbonate membrane) in starvation medium. The lower well compartment had full growth medium to stimulate cell migration across the insert membrane. After 20 h of migration, transwells were removed and cells were trypsinized from the lower surface of the membrane and counted using flow cytometry (FACSCalibur, BD Biosciences). The absolute number of cells migrating was normalized to the total cell number and values are presented as percentage of cells migrating. Values represent the average of 3 biological replicates ± standard deviation indicated as error bars (*** *P* ≤ 0.001, ** *P* ≤ 0.01, * *P* ≤ 0.05 compared to control, unpaired *t* test)

In order to evaluate the biological significance of both isoforms, we carried out a set of functional assays on MCF10A, MDA-MB-231 and MDA-MB-468 cells. All these cells are negative for ER, PR and HER2. MCF10A cells are non-transformed cells with higher genomic stability and generally higher susceptibility to perturbations of their normal growth and migratory behaviour when compared to the TNBC cell lines MDA-MB-231 and MDA-MB-468 that harbour more mutations. WST-1-based cell viability assay was used to assess the effect of overexpression of the canonical hsa-miR-140-3p and the 5’isomiR-140-3p on the cells in both cell lines. hsa-miR-140-3p did not affect the cell viability as compared to the mimic-miRNA negative controls. In contrast, 5’isomiR-140-3p caused a significant decrease in cell viability in all three cell lines (Fig. 2a).

The effects of the hsa-miR-140-3p and 5’isomiR-140-3p overexpression on the cell cycle were also tested. MCF10A, MDA-MB-231 and MDA-MB-468 cells were transfected with miRNA mimics, (Fig. 2b). In all three cell lines, 5’isomiR-140-3p overexpression resulted in a cell cycle arrest where more cells were found at the G_0_/G_1_ phase. Overexpression of the canonical hsa-miR-140-3p, however, showed no pronounced effect on the cell cycle. Analysis of baseline apoptosis in these cell lines showed no elevated activity of caspase-3/7 in 5’isomiR overexpressing cells as determined by NucView-488 caspase-3/7 assay (Biotium, Hayward, CA, USA; data not shown).

In addition, we tested the impact of overexpression of both isoforms on cell migration in a transwell-based cell migration assay. Cell numbers were normalized to a seeding control and are shown as relative values compared to control transfected cells. A decrease in cell migration was observed upon the overexpression of 5’isomiR-140-3p relative to hsa-miR-140-3p or the negative control in all three cell lines (Fig. 2c).

### miR-140-3p and its 5’isomiR have overlapping but different target spectra

**Fig. 3:**
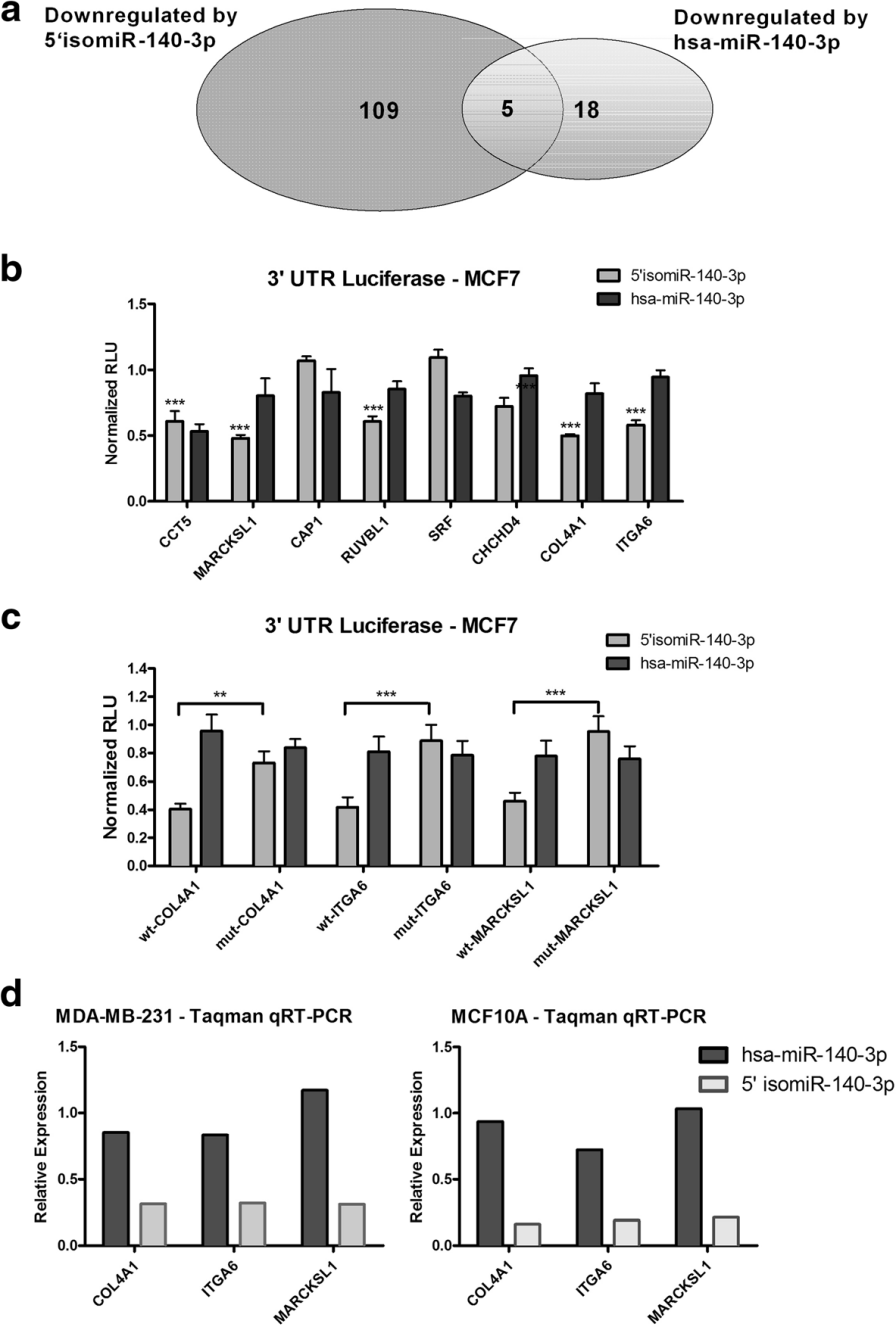
Validation of direct targeting by 3’ UTR luciferase assay and Taqman qRT-PCR. **a** Venn diagram containing genes that were found to be significantly downregulated in both MCF10A and MDA-MB-231 cells upon overexpression of has-miR-140-3p or 5’isomiR-140-3p to at least 65 % of the expression in control transfected cells. **b** and **c** MCF7 cells were transfected with the miRNA mimics and wildtype (**b**) or mutated (**c**) psiCHECK2 3’ UTR reporter plasmids as indicated. 72 h later, cells were lysed and the activity of renilla (480 nm) and firefly (560 nm) luciferase were measured. Renilla measurements were normalized to firefly and values were normalized to the negative control (mimic-ctrl2). Afterwards, values were normalized to the empty psiCHECK2 transfected with 5’ isomiR-140-3p or hsa-miR-140-3p. Bars represent the average of 6 biological replicates ± standard deviation indicated as error bars. **d** MCF10A and MDA-MB-231 cells were transfected with hsa-miR-140-3p, 5’ isomiR-140-3p or miRNA mimic negative control (mimic-ctrl2). 72 h later, cells were lysed and total mRNA was isolated and purified using RNeasy kit (Qiagen). The mRNA expression levels of the candidate genes were then assessed by Taqman qRT-PCR. Gene expression was normalized to HPRT and GAPDH housekeeping genes. Normalized gene expression is depicted as relative expression to cells transfected with mimic-ctrl2. Values represent the mean of three biological replicates (*** *P* ≤ 0.001, ** *P* ≤ 0.01, ns = non-significant compared to control, unpaired *t* test)

The 5’isomiR is shifted by one nucleotide at the 5’ end resulting in a different seed sequence, and thus is expected to have different target mRNAs. In order to examine the different spectra of target genes of the canonical miRNA and the 5’isomiR, a gene expression microarray was performed upon overexpression of both hsa-miR-140-3p and 5’isomiR-140-3p in MCF10A as well as MDA-MB-231 cells and respective negative controls in two biological replicates. Genes were considered to be downregulated by either miRNA, when their expression was reduced by at least 35 % with a significant corrected *p*-value in both cell lines. Interestingly, 109 genes were downregulated in both cell lines specifically by 5’isomiR-140-3p, whereas 18 genes were downregulated specifically in both cell lines by hsa-miR-140-3p and 5 genes were downregulated by both (Fig. 3a and Additional file 4). Of note, genes downregulated by either miR-140-3p or 5’isomiR-140-3p were significantly enriched for genes containing predicted target sites for the respective miRNA species according to TargetScan (see Additional file 5) [47].

Based on the results of the microarray analysis, we aimed to identify genes targeted only by the 5’isomiR-140-3p that might explain the tumor-suppressive phenotypes observed upon overexpression of the 5’isomiR. The 109 genes identified from the microarray were subjected to literature research with the aim of defining genes that might potentially phenocopy the viability, cell cycle and migration phenotypes seen upon the overexpression of the 5’isomiR-140-3p. The 3’ UTRs of the candidate target genes were analyzed for seed sequence matches with the 5’isomiR-140-3p. Eight putative targets, namely *SRF*, *RUVBL1*, *MARCKSL1*, *CHCHD4*, *COL4A1*, *ITGA6*, *CAP1* and *CCT5* met the aforementioned criteria. The full length 3’UTRs of the target genes were cloned into the dual luciferase reporter plasmid psiCHECK-2, a vector that utilizes Renilla luciferase as the primary reporter gene (see Additional file 6 for primer sequences). The respective reporter vectors or empty psiCHECK2 vector (as a negative control) were co-transfected with hsa-miR-140-3p or 5’isomiR-140-3p or mimic miRNA negative controls in MCF7 cells. Seventy-two hours post transfection, relative luciferase activity (renilla luciferase activity normalized to firefly luciferase activity) was measured (Fig. 3b). RLU values of target genes were normalized to the RLU of the empty psiCHECK2 vector. We identified the 3’ UTRs of *MARCKSL1*, *CHCHD4*, *COL4A1* and *ITGA6* to be specifically affected by 5’isomiR-140-3p. Moreover, 3’ UTR of *CCT5* showed a decrease in luciferase activity upon co-transfection with hsa-miR-140-3p or 5’isomiR-140-3p, indicating targeting by both forms. Therefore, it was excluded from further analyses. Additionally, *SRF* and *CAP1* were excluded from further experiments since no reduction in luciferase activity was observed compared to the empty vector.

In order to further confirm direct targeting of the candidate genes, miRNA-binding sites within the respective 3’UTRs were mutated and luciferase activity was measured. Values were normalized to the empty psiCHECK2 (Fig. 3c). Luciferase activity was rescued in all of the target genes but *CHCHD4* (data not shown). This means that the reduction observed in luciferase activity was potentially due to an indirect effect. Therefore, *CHCHD4* was omitted from further studies.

To validate downregulation of the putative target genes on mRNA level, MCF10A and MDA-MB-231 cells were transfected with hsa-miR-140-3p, 5’isomiR-140-3p or miRNA mimic negative control. The mRNA expression levels of the candidate genes were then assessed by Taqman qRT-PCR (Fig. 3d). Consistent with the previous findings from microarray and luciferase assay, a reduction in the mRNA levels of the genes *ITGA6*, *MARCKSL1* and *COL4A1* was observed.

**Fig. 4:**
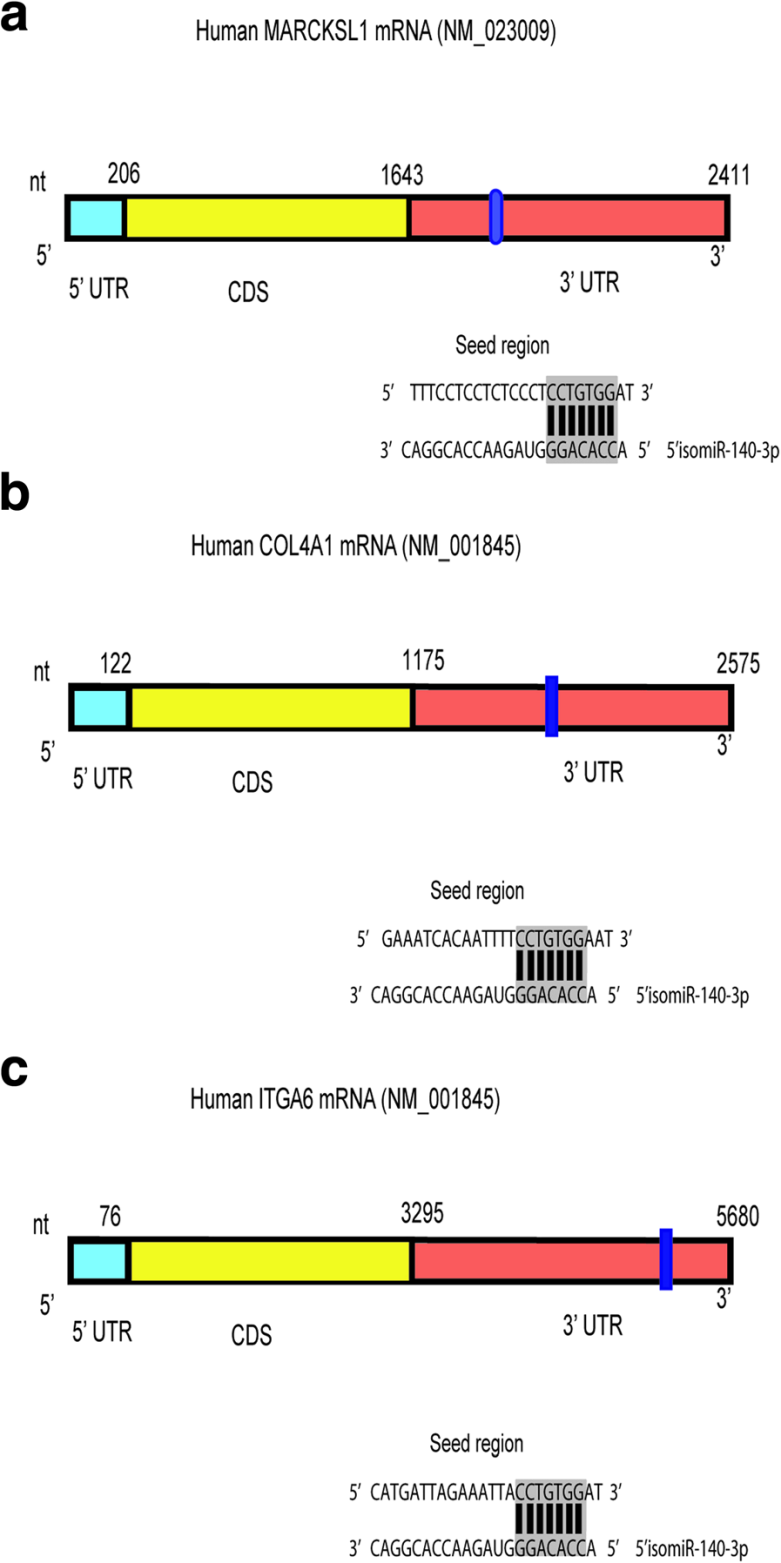
Representation of the genes and 3’UTRs of the identified target genes of 5’isomiR-140-3p, namely *MARCKSL1* (**a**), *COL4A1* (**b**) and *ITGA6* (**c**). Nucleotides that were mutated for target validation presented in Fig. 3c are highlighted

In summary, *ITGA6*, *MARCKSL1* and *COL4A1* were validated as direct targets of the 5’isomiR-140-3p and further investigated for their impact on the phenotypes observed upon overexpression of the 5’isomiR-140-3p. *COL4A1* encodes for collagen, type IV, alpha1. *MARCKSL1* encodes for a member of the myristoylated alanine-rich C-kinase substrate (MARCKS) family and *ITGA6* encodes for the integrin subunit alpha 6 and is commonly found in heterodimers known as α6β4 integrin and α6β1 integrin. Figure 4 highlights the seed regions targeted by the 5’isomiR-140-3p in the 3’UTR of each of the target genes. Nucleotides that were mutated for the experiments shown in Fig. 3c are highlighted.

### Target gene knockdown partially phenocopies overexpression of 5’isomiR-140-3p

Having identified and validated the targeting of several candidate 3’UTRs, we attempted to link the downregulated genes to the phenotypes observed upon the overexpression of the 5’isomiR-140-3p in MCF10A and/or MDA-MB-231 cells. To this end, siRNAs were used to knockdown candidate genes, and the effects on cell viability, cell cycle and cell migration were assessed. We used a set of 4 different siRNAs (numbered 1-4 according to the order numbers assigned by the Dharmacon) against each target gene. The knockdown efficiency of each individual siRNA was assessed (Additional file 7). In case of *MARCKSL1*, two siRNAs (MARCKSL1_3 and MARCKSL1_4) were found to reduce the mRNA level. In contrast, in case of *COL4A1 and ITGA6*, all four siRNAs were found to reduce its expression on the mRNA level by 70 % or more. We continued further analyses with COL4A1_2 and _4 and ITGA6_3 and _4.

**Fig. 5:**
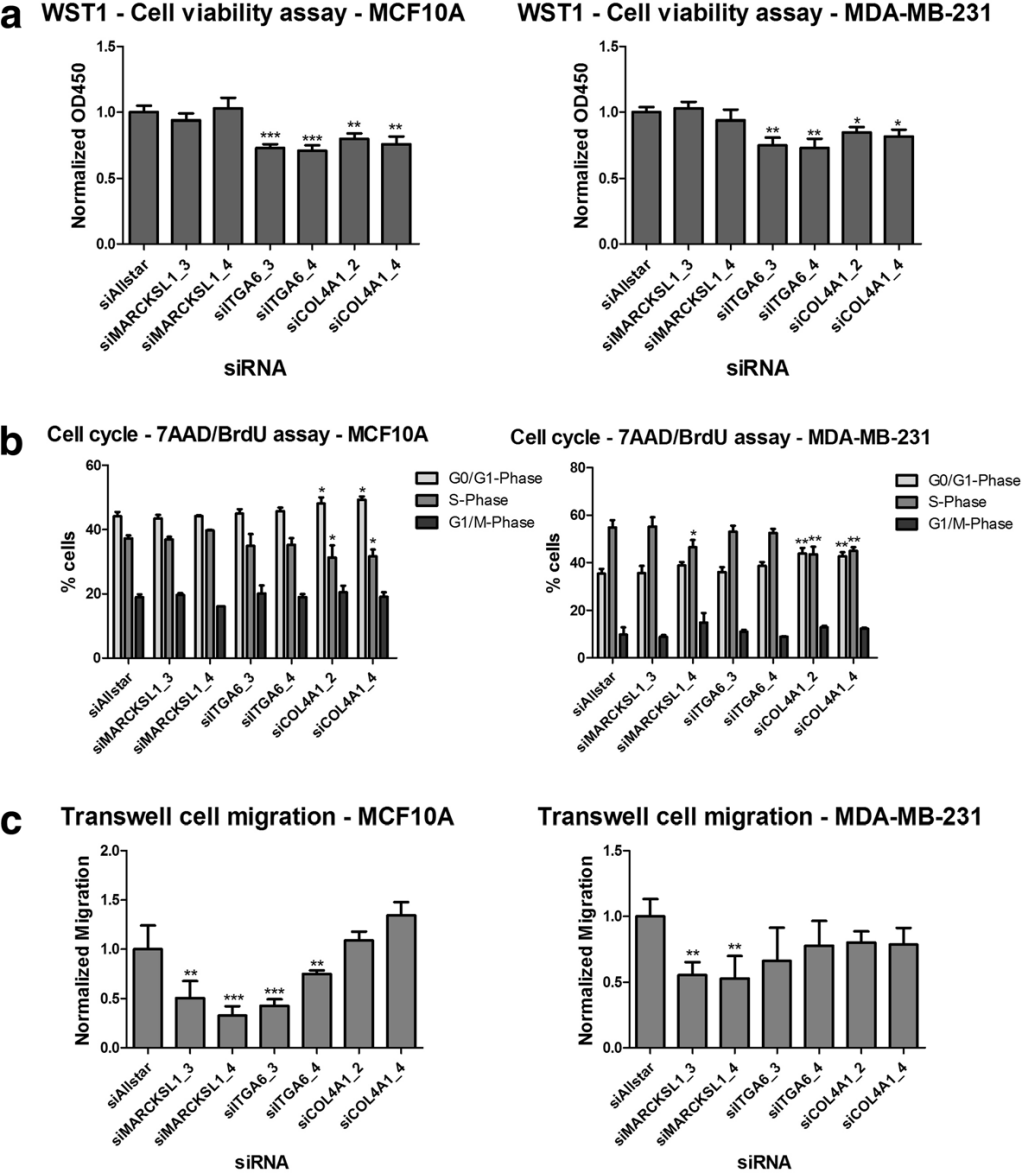
Effect of candidate genes knockdown on cell viability, cell cycle and cell migration. MCF10A and MDA-MB-231 cells were transfected with candidate siRNAs (2 individual oligos for each target gene). siAllstar was used as negative controls. **a** Cells were incubated for 72 h, then WST-1 reagent was added and the absorbance was measured at 450 nm. Results were normalized to the negative control. *COL4A1* and *ITGA6* but not *MARCKSL1* knockdown was found to decrease cell viability. Data are presented as average of 6 biological replicates ± standard deviation indicated as error bars. **b** 24 h after transfection starved in serum-free media for 24 h and allowed to re-enter the cell cycle for 24 h in full growth medium. Cells were incubated with BrdU for 30 min before fixation and lysis and stained with anti-BrdU-FITC and 7-AAD for subsequent FACS analysis. Data are presented as average and standard deviation of three biological replicates. **c** 48 h after transfection, cells were reseeded in transwell inserts (with 8.0 μm polycarbonate membrane) in starvation medium. The lower well compartment had full growth medium to stimulate cell migration across the insert membrane. After 20 h of migration, transwells were removed and cells were trypsinized from the lower surface of the membrane and counted using flow cytometry (FACSCalibur). The absolute number of cells migrating was normalized to the total cell number and values are presented as fraction of cells migrating. Values represent the average of 3 biological replicates ± standard deviation indicated as error bars (* *P* ≤ 0.05 compared to control, *t* test)

Initially, the effect of candidate gene knockdown on cell viability was evaluated. *COL4A1* and *ITGA6* knockdown was observed to phenocopy the effect of 5’isomiR-140-3p overexpression on cell viability in both cell lines and for both siRNAs (Fig. 5a). The effect was not pronounced, yet was found to be statistically significant relative to siRNA negative control siAllstar. In contrast, *MARCKSL1* knockdown did not have any effect on cell viability.

Moving on to cell cycle analysis, effects of knockdown of candidate target genes were examined. Only *COL4A1* knockdown showed cell cycle arrest with a greater fraction of cells in the G_0_/G_1_ phase and a reduction of the percentage of cells in S phase in both cell lines, similar to the 5’isomiR-140-3p (Fig 5b).

We proceeded next to examine the effect of candidate gene knockdown on cell migration using a transwell-based cell migration assay. We observed a significant decrease in cell migration upon the knockdown of *MARCKSL1* for both cell lines with both siRNAs. In addition, migration of MCF10A cells was reduced upon knockdown of *ITGA6* (Fig. 5c). The synergistic effect of these two target genes might be a partly explanation for the enhanced phenotype of the 5’isomiR in transwell migration on MCF10A cells when compared to MDA-MB-231 (see Fig. 2c).

## Discussion

IsomiR is a term coined to describe length and/or sequence variations of the same miRNA. Different mechanisms underlie the generation of isomiRs, stemming usually from the normal biogenesis pathway of miRNAs [34]. In the current work, we present the functional characterization of 5’isomiR-140-3p, where the phenotypes of different cell lines are examined upon the overexpression of the 5’isomiR-140-3p in comparison to the canonical hsa-miR-140-3p. TCGA breast cancer patients’ sequencing data revealed that the expression of both isoforms is strongly positively correlated indicating a stochastic post-transcriptional process leading to the formation of both forms [48]. Additionally, a trend of better survival among TNBC patients was observed with higher expression levels of both hsa-miR-140-3p and 5’isomiR-140-3p. This might point to a possible synergistic role of the canonical hsa-miR-140-3p and the 5’isomiR-140-3p in suppressing the growth or development of breast cancer.

The tumor suppressor role for miR-140 has been characterized and reported by Wolfson B et al., where they showed that miR-140 acts by negatively regulating Wnt, *SOX2* and *SOX9* [44]. The latter are known to be key stem cell self-renewal regulatory elements. Inhibition of miR-140 expression was found to be through an ER-mediated mechanism and/or through differential methylation of CpG islands in the miR-140 promoter region. This results in uncontrolled elevation of *SOX2*, causing an increase in stem cell populations and breast cancer initiation, progression and growth [44, 49]. Based on the hypothesis that canonical miRNAs and their functional isomiRs might synergistically target related phenotypes, we were interested in assessing the effects of overexpressing the hsa-miR-140-3p and its 5’isomiR on different cancer-associated cellular phenotypes including viability, cell cycle, and cellular migration.

We observed that, contrary to the effect of the canonical hsa-miR-140-3p, overexpression of the 5’isomiR-140-3p led to a decrease in cell viability. The latter observation was supported by cell cycle analysis, where the 5’isomiR-140-3p but not the hsa-miR-140-3p caused cell cycle arrest in G_0_/G_1_ phase evident by 7-AAD/BrdU cell cycle analysis. Co-transfection of the two forms did not result in synergistic effects on cell viability in any of these three cell lines (data not shown). Noteworthy, we found that the 5’isomiR downregulates a larger number of genes compared to the canonical miRNA annotated in miRBase. This corresponds to the observation, that the isomiR is considered to be the canonical form in several species including species closely related to humans such as gorilla.

We identified three novel direct target genes related to the observed phenotypes: *COL4A1*, *ITGA6* and *MARCKSL1. COL4A1* encodes for collagen, type IV, alpha1. Type IV collagen is a main component of basement membranes, where molecules attach to each other forming complex protein networks. These networks help the basement membranes interact with nearby cells, playing a role in cell movement or migration, cell survival and proliferation, and cell differentiation [49]. We observed that knockdown of *COL4A1* caused a slight reduction in cell viability, cell cycle arrest in the G0/G1 phase and decrease in cellular migration through transwells. Previous studies pointed to the effect of *COL4A1* in tumor progression and metastasis. Chen et al. observed a change in morphology of the skin cancer cells upon knocking down the *COL4A1* gene [50]. They also noted that knocking down *COL4A*1 conferred the cells with higher levels of elasticity and lower motility.

*ITGA6* is the gene encoding for the α6 subunit of integrins and forms heterodimers preferentially with the β4 and β1 subunits. Integrins are a group of proteins that regulate the cell-cell adhesion as well as the cell-matrix adhesion. They also transmit chemical signals that modulate cell growth and can alter the activity of certain genes [51]. Similar to *COL4A1*, we observed that knockdown of *ITGA6* caused a reduction in cell viability and in MCF10A cells a decrease in cellular migration through transwells. In the study of hepatocellular carcinoma (HCC), it was shown that Integrin α6 can mediate metastasis, and that it could be used as a therapeutic target for improved patients’ survival. Knockdown of *ITGA6* using shRNA was found to inhibit the proliferation and metastasis of HCC cells though PI3K/AKT and MAPK/ERK, where p-ERK and p-AKT were reduced by shRNA targeting integrin α6 [52]. The effects of 5’isomiR-140-3p overexpression on the signaling molecules downstream of the collagen/Integrin signaling pathway remain to be studied. Additionally, in esophageal squamous cell carcinoma (ESCC), *ITGA6* was found to be highly expressed. In agreement with the results reported in HCC, *in vitro* knockdown of *ITGA6* in ESCC cells resulted in inhibition of cell proliferation, invasion and colony formation [53]. Given the tight connection between integrins and collagens, their combinatorial downregulation by the 5’isomiR might be a reason for the reduced migratory potential of cells overexpressing the 5’isomiR.

*MARCKSL1* encodes for a member of the myristoylated alanine-rich C- kinase substrate (MARCKS) family. This family of proteins functions in cytoskeletal regulation, protein kinase C signaling and calmodulin signaling. MARCKSL1 protein affects the formation of the intermediate junction (also called adherens junction or belt desmosome), which is a type of cell-cell junctions occurring in epithelial as well as endothelial cells [54, 55]. Our experiments revealed a significant reduction in cellular migration across the transwells upon the knockdown of *MARCKSL1*. Consistent with these results, Jonsdottir et al. studied the prognostic value of *MARCKSL1* in lymph node-negative breast cancer patients. Among different prognosticators studied (age, tumor diameter, grade, estrogen receptor, and proliferation), MARCKSL1 protein expression was the strongest prognosticator. Patients with high MARCKSL1 expression showed a 44 % survival versus 88 % in patients with low expression at 15-year follow-up. Moreover, distant metastasis free survival was found to be significantly higher in patients with low MARCKSL1 protein expression (78 %), compared to 45 % survival for patients with high expression [56]. Additionally, in a separate study that involved breast cancer metastasis to the bone, *MARCKSL1* was reported to be upregulated. Björkblom et al. searched the *In Silico* Transcriptomics (IST) database for *MARCKSL1* expression data. They found that in normal tissues, *MARCKSL1* mRNA was highly expressed in the central nervous system, testis, ovary, and lymphatic organs. In addition to breast cancer, there was significant upregulation of *MARCKSL1* in lung cancer, rhabdomyosarcoma, leiomyosarcoma, prostate cancer and uterine cancer [55].

Counter-intuitively, knockdown of *MARCKSL1* in different tissue contexts, specifically the neural tissue, and prostate cancer cell line (PC-3) resulted in an abrupt increase in migration. This was explained by the fact that activation of MARCKSL1 through phosphorylation leads to its interaction with actin, reducing actin turnover in cells and retarding cell migration [55]. Hence, it seems to depend on the cellular context and the phosphorylation status of MARCKSL1 whether its downregulation results in increased or decreased migration of the cells.

## Conclusions

**Fig. 6:**
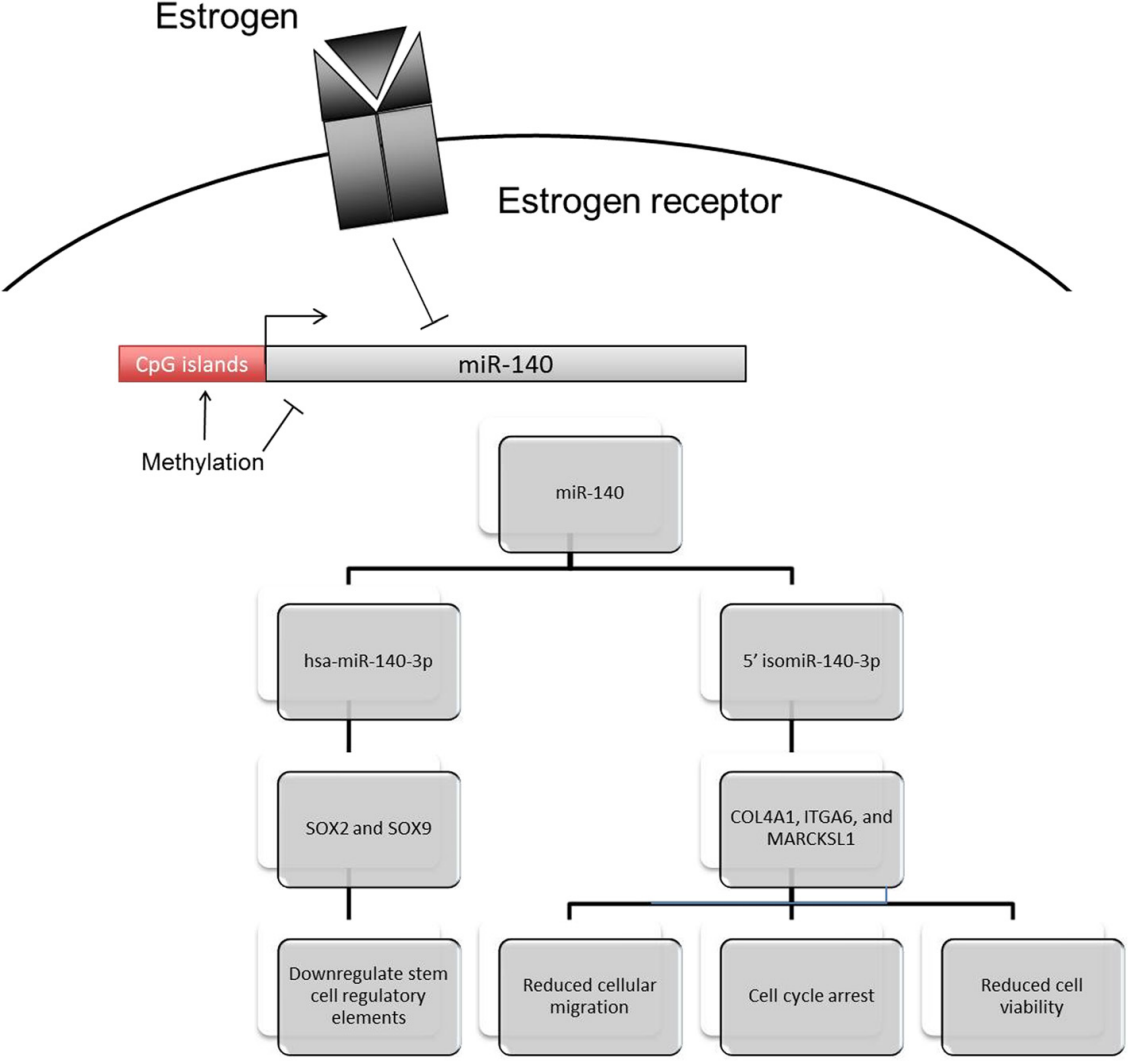
Schematic diagram summarizing the role of the miR-140 in the course of breast cancer development. miR-140 expression is inhibited by the action of estrogen receptor or through differential methylation of CpG islands in its promoter region. Upon its expression, two different isoforms are formed that act synergistically to suppress the growth and development of breast cancer. The canonical hsa-miR-140-3p acts by negatively regulating the Wnt, *SOX2* and *SOX9* [44]. The latter are known to be key stem cell self-renewal regulatory elements. Inhibition of miR-140 expression results in uncontrolled elevation of *SOX2*, causing an increase in stem cell populations and breast cancer initiation, progression and growth. The 5’isomiR-140-3p acts by reducing cellular viability, cellular migration and arresting the cell cycle in the G0/G1 phase through targeting *COL4A1*, *ITGA6*, and *MARCKSL1*

In conclusion, in this work we present the characterization of 5’isomiR-140-3p. Its overexpression in breast cancer cells resulted in phenotypes counteracting the progression of cancer including reduction in cell viability, cell cycle arrest and inhibition of migration. Moreover, we were able to identify and validate novel targets for the 5’isomiR-140-3p; *COL4A1*, *ITGA6* and *MARCKSL*. Finally, we have shown that knocking down these genes could partially phenocopy the effects of the 5’isomiR-140-4p overexpression. *COL4A1* and *ITGA6* knockdown led to less cell viability and cell cycle arrest, while *MARCKSL1* knockdown resulted in a decrease in the migratory potential of cells. Figure 6 represents a summary of our understanding to the regulation of the miR-140 expression and its targets.

This study shows – to our knowledge – the first example on the functional discrepancy of the two highly expressed isoforms a miRNA in the context of human cancer. Despite the difference in their regulatory roles, both the canonical miRNA and the 5’isomiR work in a tumor suppressor direction. It’s worth mentioning that the mechanism underlying the regulation of the canonical or the 5’isomiR expression is not yet fully understood. Interestingly, we found that the ratio of the canonical miRNA and the 5’isomiR was significantly shifted towards to canonical form when comparing normal breast tissue with breast cancer tissue data, both from the TCGA dataset. An open question to be asked here is whether and how the cell can decide for or control which variant of the miRNA to express at which circumstances.

Lastly, with isomiRs in picture, the study of miRNAome is way more complex than previously thought. A growing number of reports point to the biological significance of at least some isomiRs. One nucleotide shift changes the spectrum of targeted genes, changing thereby the functional regulatory role of the miRNA. Through further characterization of the canonical miR-140-3p and the 5’ismoiR-140-3p and other miRNA/5’isomiR pairs, more insights will be gained into the carcinogenesis of breast cancers. This will potentially provide one more tool to be used by clinicians in diagnosis or treatment.

## Methods

### Cell culture and reagents

MCF10A, MDA-MB-231, MDA-MB-468 and MCF-7 cell lines were obtained from ATCC (Manassas, VA, USA) in 2009 and 2010. Cell lines were verified using the cell line authentication service at the DKFZ Core Facility by Multiplex human cell authentication in 2011 (23). Cells were incubated in a humidified incubator at 37 °C and 5 % CO_2._ MCF10A cells were cultured in DMEM F12 medium (5 % Horse serum, 20 ng/ml EGF (BD Biosciences, Bedford, MA, USA), 0.5 μg/ml Hydrocortisone (Sigma-Aldrich, Saint-Louis, MO, USA), 100 ng/ml Choleratoxin (Sigma-Aldrich, Saint-Louis, MO, USA), 0.01 mg/ml bovine insulin (Sigma-Aldrich, Saint-Louis, MO, USA), 50 units/ml penicillin, 50 μg/ml streptomycin sulfate). MDA-MB-231 and MDA-MB-468 cells were maintained in RPMI1640 medium (10 % FBS, 1 % L-glutamine, 1 % nonessential amino acids, 50 units/ml penicillin, 50 μg/ml streptomycin sulfate). MCF-7 cells were cultured in MEM medium (10 % FBS, 1 % nonessential amino acids, 1 % L-glutamine, 50 units/ml penicillin, 50 μg/ml streptomycin sulfate). Cell culture media and reagents were obtained from Invitrogen.

### Transfections

All transfections were performed using Lipofectamine 2000 (Invitrogen) according to the manufacturer’s protocol. miRNA mimics were purchased from Ambion (Life technologies). The mature sequences for hsa-miR-140-3p and 5’isomiR-140-3p are 5’UACCACAGGGUAGAACCACGG 3’ and 5’ ACCACAGGGUAGAACCACGGAC 3’ respectively. miRNA negative controls were obtained from Dharmacon (Lafayette, CO). siRNAs against *COL4A1*, *ITGA6*, and *MARCKSL1* were purchased from Dharmacon as set of four individual siRNAs per gene. Two of them were used for phenotypic analyses. siAllStar non-targeting control was obtained from Qiagen (Hilden, Germany). siRNAs and miRNA mimics were used at a final concentration of 30 nM.

### Microarray-based analysis of deregulated genes

MCF10A and MDA-MB-231 cells were seeded in 6-well plates and transfected with miRNA mimics for hsa-miR-140-3p and 5’isomiR-140-3p in two biological replicates, each, as well as with one replicate of mimic-ctrl1 and mimic-ctrl2. 48 h after transfection, RNA was extracted using the RNeasy kit (Qiagen, Hilden, Germany) and submitted to the Microarray unit of the Genomics-Proteomics Core Facility of the German Cancer Research Center. Genes were considered to be downregulated by the canonical miRNA or the 5’isomiR, respectively, if they showed an expression of less than 65 % of the expression in control transfected cells and the corrected p-value was below 0.05. Results from the microarray were uploaded to GEO (GSE74539).

### Luciferase reporter assays

To validate direct targeting of miRNAs, 100 ng/well psiCHECK2 vectors (Promega, Madison, WI, USA), containing the respective 3’UTRs, were co-transfected with mimic miRNAs in MCF-7 cells. 48 h after transfection *Renilla* and firefly luciferase activities were determined as previously described using a luminometer (Tecan, Linz, Austria) [57]. Mutations within each of the predicted target sites of *the* 3’-UTRs were generated by site-directed mutagenesis using Quikchange Lightning kit (Aglient Techonologies). Primers are listed in Additional file 6.

### RNA isolation and real-time PCR

Total RNA was isolated from cells using the RNeasy mini kit (Qiagen) according to the manufacturer’s instructions. Reverse transcription of mRNAs into cDNA was done using the Transcriptor Reverse Transcriptase kit (Roche). The qRT-PCR reactions for target genes were performed using the ABI Prism 7900HT sequence detection system (Applied Biosystems, Weiterstadt, Germany), using probes from the Universal Probe Library (Roche Diagnostics). For normalization of the mRNA analysis, HPRT1 and GAPDH were used as housekeeping genes.

### WST-1 based cell viability assay

5 × 10^3^ MCF-10A, MDA-MB-468 or MDA-MB-231 cells/well were seeded into 96-well plates. After 24 h, cells were transfected with the respective miRNAs or siRNAs. Transfections were performed in 6 replicates. 72 h later, 10 μL/well WST-1 reagent were added and the absorbance of the plate was measured at 450 nm using the TECAN plate reader (Infinite® M200). As medium blank, 10 μL of WST-1 reagent was added to a well containing only medium without cells and the absorbance of this well was subtracted from all other values obtained.

### Cell cycle analysis

Cells were seeded as a single cell suspension with 1.5 × 10^5^ cells/well into 6-well plates and transfected as described above. 48 h after transfection, the cells were incubated with BrdU for 30 min before fixation with Cytofix/Cytoperm (BD Biosciences, Heidelberg, Germany) according to the manufacturer’s recommendations. BrdU was stained with a FITC-conjugated anti-BrdU antibody (BD Biosciences, Heidelberg, Germany) and DNA was stained with 7-AAD. Samples were analyzed using a FACSCalibur (Becton-Dickenson) and their distributions in the distinct cell cycle phases G_0_/G_1_, S and G_2_/M phases was assessed based on their fluorescence intensity for both dyes.

### Transwell-based cell migration assay

MCF10A cells were transfected with 30 nM siRNAs or miRNAs in 6-well plates as described above. 48 h after transfection, cells were detached and 2 × 10^5^ cells were seeded in starvation medium into transwell inserts with 8.0 μm pore-sized membranes in the 24-well plates format (Corning). Full growth medium was used as chemoattractant in the lower chamber. To control for the number of cells, cells from the same suspension were seeded into poly-L-lysine coated 0.72 μm glass bottom square well MatriPlates. After 6 h (to allow for cell attachment), cells were stained with Hoechst-33258 and counted using Olympus ScanR microscope. In the transwells, cells were allowed to migrate for 20 h and the number of migrating cells was determined by flow cytometry using a FACSCalibur (Becton-Dickenson). For that purpose, cells attached to the lower side of the membrane were trypsinized and transferred into FACS-tubes. Cells were centrifuged, washed once with PBS and resuspended in 150 μl PBS. Cells were counted for a total of 2 min at high flow rate. The number of cells migrating was normalized to the total cell number from the counting control and values were presented as fraction of cells migrating.

### Analysis of small RNA sequencing of breast cancer cell lines

Small RNA sequencing data distributed during the Illumina iDEA challenge was submitted to ArrayExpress (E-MTAB-4539) with kind permission by Illumina; details on the experimental setup can be found in the respective metadata. Briefly, the data had been generated by Illumina on a Gene Analyzer instrument using total RNA from eight commonly used breast cancer cell lines. Adapters were trimmed using trimmomatic and bowtie was used for mapping of the sequences to the genome (GRCh38) without allowing for any mismatches. IsomiR notation was based on miRBase version 21 and reads were mapped to the specific isomiR using bedtools intersect.

We analyzed the data for the presence of highly abundant miRNAs with associated highly abundant 5’isomiRs using an average expression over all cell lines of 100 rpm and a ratio of isomiR to miR of at least 1:5 as threshold to define potentially interesting pairs.

To do so, we did not distinguish variants at the 3’end of both species, treating all 3’isomiRs with a length of 18-24 nucleotides as miRNA or 5’isomiR, respectively, depending on their 5’ end. Using the notation suggested by Loher and colleagues [27], we refer for example to all miRNA species between hsa-miR-140-3p 0|-3 and 0| + 3 (in Table 1 simplified as 0|x) as canonical hsa-miR-140-3p, whereas all species between hsa-miR-140-3p +1|-2 to +1| + 3 (in Table 1 simplified as +1|x) are considered as 5’isomiR-140-3p.

### Patient data analyses

Expression values of hsa-miR-140-3p and the 5’isomiR-140-3p were extracted from the TCGA breast cancer dataset (accessed August 2013) and correlated with clinical parameters such as ER- or metastasis status [46]. The reads for miR and isomiR as well as the clinical parameters are provided as Additional file 2. Survival analyses were performed using GraphPad Prism verson 6.0; the upper and the lower quartiles of expression were considered as ‘high’ and ‘low’, respectively.

### Statistical analyses

Data are presented as mean ± S.D. Samples were analyzed by two-tailed unpaired Student’s t test, unless otherwise mentioned, and *p* values < 0.05 were considered as being statistically significant. *p* values < 0.05, < 0.01, and < 0.001 are indicated with one, two, and three asterisks, respectively. Data obtained from breast cancer patient samples were analyzed using GraphPad Prism verson 6.0.

## Abbreviations

COL4A1, collagen, type IV, alpha1; ER, estrogen receptor; FACS, Fluorescence-activated cell sorting; HER2, human epidermal growth factor receptor 2; ITGA6, integrin alpha 6; MARCKSL1, myristoylated alanine-rich C-kinase substrate like 1; miRNA, microRNA; PBS, phosphate buffered saline; PR, progesterone receptor; RISC, RNA-induced silencing complex; siRNA, small interfering RNA; TCGA, The Cancer Genome Atlas; UTR, untranslated region

## Additional files

Additional file 1: miRNA seq dataset used to identify highly abundant miRNAs that have abundant 5’isomiRs. (XLSX 3320 kb) Additional file 2: Clinical data and miR/isomiR expression of patients from the TCGA breast cancer cohort [48]. (XLS 7116 kb) Additional file 3: Relative abundance of 3’isomiRs of miR-140-3p and 5’isomiR-140-3p in the TCGA breast cancer patient cohort. The table lists the genomic coordinates, the notation of the isomiR and the average expression of each individual form (in rpm). (XLSX 9 kb) Additional file 4: Genes deregulated by hsa-miR-140-3p, 5’isomiR-140-3p or both in both MCF10A and MDA-MB-231 cells. This table contains the genes which are downregulated in both cell lines by either hsa-miR-140-3p, 5’isomiR-140-3p or both by at least 35 % and with a significant corrected *p*-value. These genes are summarized in the Venn diagram in Fig. 2a. In addition, the genes that are upregulated in both cell lines by at least 65 % and with a significant corrected *p*-value by either has-miR-140-3p, 5’isomiR-140-3p or both are also listed in this table. Values are reported as log2 fold changes and Benjamini-Hochberg corrected *p*-values. (XLS 74 kb) Additional file 5: Venn Diagrams of predicted and downregulated genes for both miRNA species. Predicted targets according to TargetScan irrespective of site conservation and downregulated genes as listed in Additional file 3 for miR-140-3p (A) and 5’isomiR-140-3p (B) are depicted. For both miRNA species, downregulated genes are significantly enriched for predicted targets of the respective miRNA (*p* < 0.001 as determined by Chi-square test). (JPG 62 kb) Additional file 6: List of primers and probes. This file contains all primer sequences used for cloning and mutagenesis of the luciferase reporter plasmids, as well as the UPL primers and probes used for qRT-PCR. (DOCX 17 kb) Additional file 7: Efficiency of target gene knockdown on the mRNA level using Taqman qRT-PCR. MCF10A cells were transfected with different siRNAs or siRNA negative control (siAllstar). 72 h later, cells were lysed and total mRNA was isolated and purified using RNeasy kit (Qiagen). The mRNA expression levels of the candidate genes were then assessed by Taqman qRT-PCR. Gene expression was normalized to HPRT and GAPDH housekeeping genes. Normalized gene expression is depicted as relative expression to cells transfected with siAllstar. Values represent the mean of three technical replicates. (JPG 350 kb)

## References

[1] Kayani M, Kayani MA, Malik FA, Faryal R (2011). Role of miRNAs in breast cancer. Asian Pac J Cancer Prev.

[2] Weigelt B, Peterse JL, van ’t Veer LJ (2005). Breast cancer metastasis: markers and models. Nat Rev Cancer.

[3] Sorlie T, Tibshirani R, Parker J, Hastie T, Marron JS, Nobel A, Deng S, Johnsen H, Pesich R, Geisler S (2003). Repeated observation of breast tumor subtypes in independent gene expression data sets. Proc Natl Acad Sci U S A.

[4] Sorlie T, Perou CM, Tibshirani R, Aas T, Geisler S, Johnsen H, Hastie T, Eisen MB, van de Rijn M, Jeffrey SS (2001). Gene expression patterns of breast carcinomas distinguish tumor subclasses with clinical implications. Proc Natl Acad Sci U S A.

[5] Perou CM, Sorlie T, Eisen MB, van de Rijn M, Jeffrey SS, Rees CA, Pollack JR, Ross DT, Johnsen H, Akslen LA (2000). Molecular portraits of human breast tumours. Nature.

[6] Eroles P, Bosch A, Perez-Fidalgo JA, Lluch A (2012). Molecular biology in breast cancer: intrinsic subtypes and signaling pathways. Cancer Treat Rev.

[7] Seidman AD, Berry D, Cirrincione C, Harris L, Muss H, Marcom PK, Gipson G, Burstein H, Lake D, Shapiro CL (2008). Randomized phase III trial of weekly compared with every-3-weeks paclitaxel for metastatic breast cancer, with trastuzumab for all HER-2 overexpressors and random assignment to trastuzumab or not in HER-2 nonoverexpressors: final results of Cancer and Leukemia Group B protocol 9840. J Clin Oncol.

[8] Vogel CL, Cobleigh MA, Tripathy D, Gutheil JC, Harris LN, Fehrenbacher L, Slamon DJ, Murphy M, Novotny WF, Burchmore M (2002). Efficacy and safety of trastuzumab as a single agent in first-line treatment of HER2-overexpressing metastatic breast cancer. J Clin Oncol.

[9] Dent R, Trudeau M, Pritchard KI, Hanna WM, Kahn HK, Sawka CA, Lickley LA, Rawlinson E, Sun P, Narod SA (2007). Triple-negative breast cancer: clinical features and patterns of recurrence. Clin Cancer Res.

[10] Cheang MC, Chia SK, Voduc D, Gao D, Leung S, Snider J, Watson M, Davies S, Bernard PS, Parker JS (2009). Ki67 index, HER2 status, and prognosis of patients with luminal B breast cancer. J Natl Cancer Inst.

[11] Radojicic J, Zaravinos A, Vrekoussis T, Kafousi M, Spandidos DA, Stathopoulos EN (2011). MicroRNA expression analysis in triple-negative (ER, PR and Her2/neu) breast cancer. Cell Cycle.

[12] Esquela-Kerscher A, Slack FJ (2006). Oncomirs - microRNAs with a role in cancer. Nat Rev Cancer.

[13] Gregory RI, Yan KP, Amuthan G, Chendrimada T, Doratotaj B, Cooch N, Shiekhattar R (2004). The Microprocessor complex mediates the genesis of microRNAs. Nature.

[14] Lee Y, Jeon K, Lee JT, Kim S, Kim VN (2002). MicroRNA maturation: stepwise processing and subcellular localization. EMBO J.

[15] Lee YS, Dutta A (2009). MicroRNAs in cancer. Annu Rev Pathol.

[16] Curtis C, Shah SP, Chin SF, Turashvili G, Rueda OM, Dunning MJ, Speed D, Lynch AG, Samarajiwa S, Yuan Y (2012). The genomic and transcriptomic architecture of 2,000 breast tumours reveals novel subgroups. Nature.

[17] Croce CM (2009). Causes and consequences of microRNA dysregulation in cancer. Nat Rev Genet.

[18] Lu J, Getz G, Miska EA, Alvarez-Saavedra E, Lamb J, Peck D, Sweet-Cordero A, Ebert BL, Mak RH, Ferrando AA (2005). MicroRNA expression profiles classify human cancers. Nature.

[19] Di Leva G, Croce CM (2013). miRNA profiling of cancer. Curr Opin Genet Dev.

[20] Volinia S, Calin GA, Liu CG, Ambs S, Cimmino A, Petrocca F, Visone R, Iorio M, Roldo C, Ferracin M (2006). A microRNA expression signature of human solid tumors defines cancer gene targets. Proc Natl Acad Sci U S A.

[21] Iorio MV, Croce CM (2012). MicroRNA dysregulation in cancer: diagnostics, monitoring and therapeutics. A comprehensive review. EMBO Mol Med.

[22] Kasinski AL, Slack FJ (2011). Epigenetics and genetics. MicroRNAs en route to the clinic: progress in validating and targeting microRNAs for cancer therapy. Nat Rev Cancer.

[23] Landgraf P, Rusu M, Sheridan R, Sewer A, Iovino N, Aravin A, Pfeffer S, Rice A, Kamphorst AO, Landthaler M (2007). A mammalian microRNA expression atlas based on small RNA library sequencing. Cell.

[24] Wyman SK, Knouf EC, Parkin RK, Fritz BR, Lin DW, Dennis LM, Krouse MA, Webster PJ, Tewari M (2011). Post-transcriptional generation of miRNA variants by multiple nucleotidyl transferases contributes to miRNA transcriptome complexity. Genome Res.

[25] Lee LW, Zhang S, Etheridge A, Ma L, Martin D, Galas D, Wang K (2010). Complexity of the microRNA repertoire revealed by next-generation sequencing. RNA.

[26] Cloonan N, Wani S, Xu Q, Gu J, Lea K, Heater S, Barbacioru C, Steptoe AL, Martin HC, Nourbakhsh E (2011). MicroRNAs and their isomiRs function cooperatively to target common biological pathways. Genome Biol.

[27] Loher P, Londin ER, Rigoutsos I (2014). IsomiR expression profiles in human lymphoblastoid cell lines exhibit population and gender dependencies. Oncotarget.

[28] Telonis AG, Loher P, Jing Y, Londin E, Rigoutsos I (2015). Beyond the one-locus-one-miRNA paradigm: microRNA isoforms enable deeper insights into breast cancer heterogeneity. Nucleic Acids Res.

[29] Chan YT, Lin YC, Lin RJ, Kuo HH, Thang WC, Chiu KP, Yu AL (2013). Concordant and discordant regulation of target genes by miR-31 and its isoforms. PLoS One.

[30] Wu H, Ye C, Ramirez D, Manjunath N (2009). Alternative processing of primary microRNA transcripts by Drosha generates 5’ end variation of mature microRNA. PLoS One.

[31] Liu N, Abe M, Sabin LR, Hendriks GJ, Naqvi AS, Yu Z, Cherry S, Bonini NM (2011). The exoribonuclease Nibbler controls 3’ end processing of microRNAs in Drosophila. Curr Biol.

[32] Han BW, Hung JH, Weng Z, Zamore PD, Ameres SL (2011). The 3’-to-5’ exoribonuclease Nibbler shapes the 3’ ends of microRNAs bound to Drosophila Argonaute1. Curr Biol.

[33] Martin G, Keller W (2007). RNA-specific ribonucleotidyl transferases. RNA.

[34] Neilsen CT, Goodall GJ, Bracken CP (2012). IsomiRs--the overlooked repertoire in the dynamic microRNAome. Trends Genet.

[35] Nishikura K (2010). Functions and regulation of RNA editing by ADAR deaminases. Annu Rev Biochem.

[36] Newman MA, Mani V, Hammond SM (2011). Deep sequencing of microRNA precursors reveals extensive 3’ end modification. RNA.

[37] Burroughs AM, Ando Y, de Hoon MJ, Tomaru Y, Nishibu T, Ukekawa R, Funakoshi T, Kurokawa T, Suzuki H, Hayashizaki Y (2010). A comprehensive survey of 3’ animal miRNA modification events and a possible role for 3’ adenylation in modulating miRNA targeting effectiveness. Genome Res.

[38] Miyaki S, Sato T, Inoue A, Otsuki S, Ito Y, Yokoyama S, Kato Y, Takemoto F, Nakasa T, Yamashita S (2010). MicroRNA-140 plays dual roles in both cartilage development and homeostasis. Genes Dev.

[39] Song B, Wang Y, Xi Y, Kudo K, Bruheim S, Botchkina GI, Gavin E, Wan Y, Formentini A, Kornmann M (2009). Mechanism of chemoresistance mediated by miR-140 in human osteosarcoma and colon cancer cells. Oncogene.

[40] Iorio MV, Visone R, Di Leva G, Donati V, Petrocca F, Casalini P, Taccioli C, Volinia S, Liu CG, Alder H (2007). MicroRNA signatures in human ovarian cancer. Cancer Res.

[41] Sand M, Skrygan M, Sand D, Georgas D, Hahn SA, Gambichler T, Altmeyer P, Bechara FG (2012). Expression of microRNAs in basal cell carcinoma. Br J Dermatol.

[42] Zou MX, Huang W, Wang XB, Lv GH, Li J, Deng YW (2014). Identification of miR-140-3p as a marker associated with poor prognosis in spinal chordoma. Int J Clin Exp Pathol.

[43] Rakoczy J, Fernandez-Valverde SL, Glazov EA, Wainwright EN, Sato T, Takada S, Combes AN, Korbie DJ, Miller D, Grimmond SM (2013). MicroRNAs-140-5p/140-3p modulate Leydig cell numbers in the developing mouse testis. Biol Reprod.

[44] Wolfson B, Eades G, Zhou Q (2014). Roles of microRNA-140 in stem cell-associated early stage breast cancer. World J Stem Cells.

[45] Ha M, Kim VN (2014). Regulation of microRNA biogenesis. Nat Rev Mol Cell Biol.

[46] Cancer Genome Atlas N (2012). Comprehensive molecular portraits of human breast tumours. Nature.

[47] Agarwal V, Bell GW, Nam JW, Bartel DP. Predicting effective microRNA target sites in mammalian mRNAs. eLife. 2015;4. doi:10.7554/eLife.05005. 10.7554/eLife.05005 PMC4532895 26267216

[48] Cancer Genome Atlas Network. Comprehensive molecular portraits of human breast tumours. Nature. 2012;490(7418):61-70. 10.1038/nature11412 PMC3465532 23000897

[49] Breedveld G, de Coo IF, Lequin MH, Arts WF, Heutink P, Gould DB, John SW, Oostra B, Mancini GM (2006). Novel mutations in three families confirm a major role of COL4A1 in hereditary porencephaly. J Med Genet.

[50] Chen SY, Lin JS, Yang BC (2014). Modulation of tumor cell stiffness and migration by type IV collagen through direct activation of integrin signaling pathway. Arch Biochem Biophys.

[51] Hynes RO (1992). Integrins: versatility, modulation, and signaling in cell adhesion. Cell.

[52] Lv G, Lv T, Qiao S, Li W, Gao W, Zhao X, Wang J (2013). RNA interference targeting human integrin alpha6 suppresses the metastasis potential of hepatocellular carcinoma cells. Eur J Med Res.

[53] Kwon J, Lee TS, Lee HW, Kang MC, Yoon HJ, Kim JH, Park JH (2013). Integrin alpha 6: a novel therapeutic target in esophageal squamous cell carcinoma. Int J Oncol.

[54] Yarmola EG, Edison AS, Lenox RH, Bubb MR (2001). Actin filament cross-linking by MARCKS: characterization of two actin-binding sites within the phosphorylation site domain. J Biol Chem.

[55] Bjorkblom B, Padzik A, Mohammad H, Westerlund N, Komulainen E, Hollos P, Parviainen L, Papageorgiou AC, Iljin K, Kallioniemi O (2012). c-Jun N-terminal kinase phosphorylation of MARCKSL1 determines actin stability and migration in neurons and in cancer cells. Mol Cell Biol.

[56] Jonsdottir K, Zhang H, Jhagroe D, Skaland I, Slewa A, Bjorkblom B, Coffey ET, Gudlaugsson E, Smaaland R, Janssen EA (2012). The prognostic value of MARCKS-like 1 in lymph node-negative breast cancer. Breast Cancer Res Treat.

[57] Keklikoglou I, Koerner C, Schmidt C, Zhang JD, Heckmann D, Shavinskaya A, Allgayer H, Guckel B, Fehm T, Schneeweiss A (2012). MicroRNA-520/373 family functions as a tumor suppressor in estrogen receptor negative breast cancer by targeting NF-kappaB and TGF-beta signaling pathways. Oncogene.

