# Supplementary figures and images for: The highly expressed 5’isomiR of hsa-miR-140-3p contributes to the tumor-suppressive effects of miR-140 by reducing breast cancer proliferation and migration
Source: BMC Genomics. 2016 Aug 8;17:566. doi: 10.1186/s12864-016-2869-x (PMC4977694; doi:10.1186/s12864-016-2869-x)

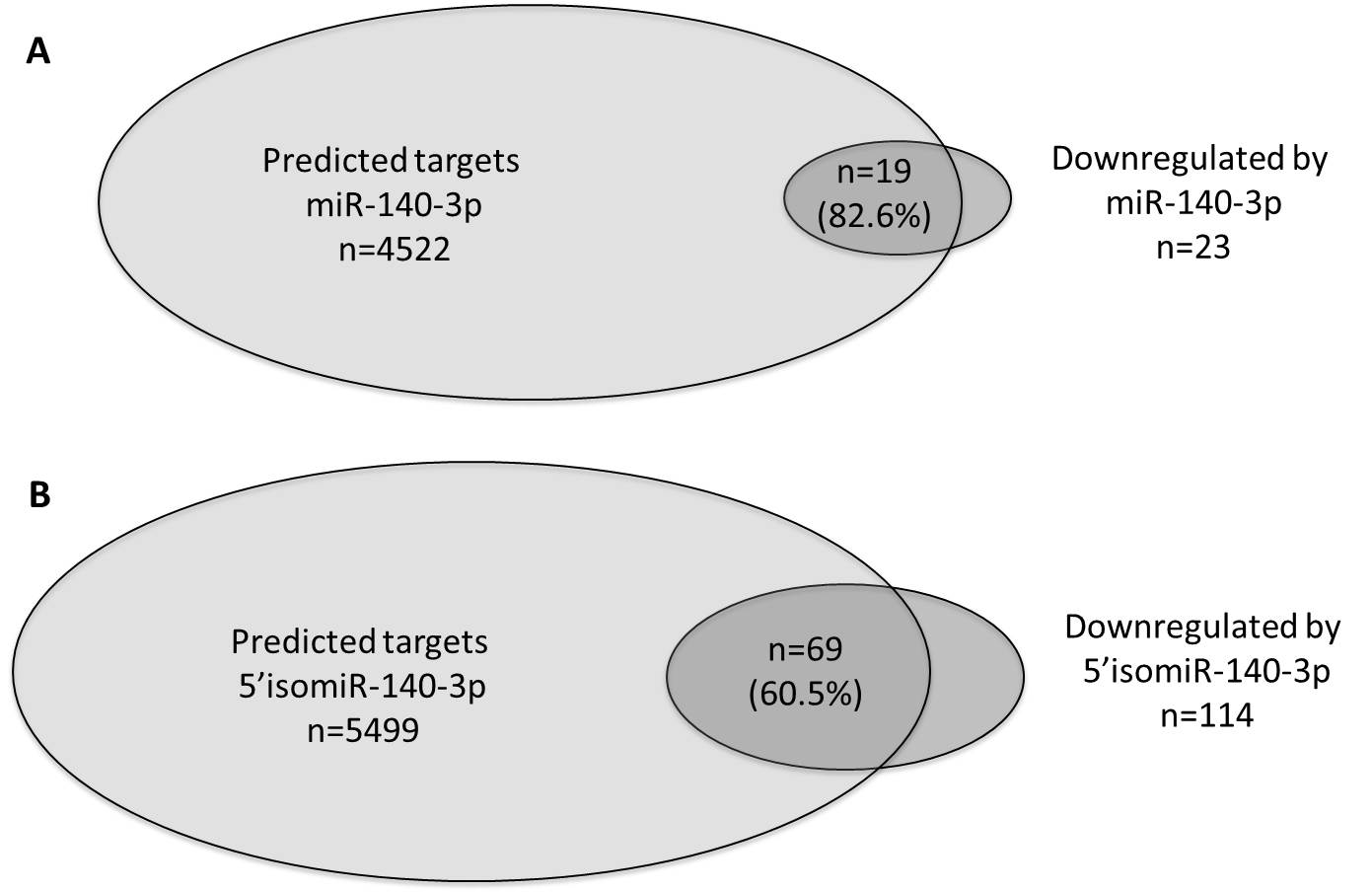

Supplement: Additional file 5: — Venn Diagrams of predicted and downregulated genes for both miRNA species. Predicted targets according to TargetScan irrespective of site conservation and downregulated genes as listed in Additional file 3 for miR-140-3p (A) and 5’isomiR-140-3p (B) are depicted. For both miRNA species, downregulated genes are significantly enriched for predicted targets of the respective miRNA (p < 0.001 as determined by Chi-square test). (JPG 62 kb) [file 12864_2016_2869_MOESM5_ESM.jpg]

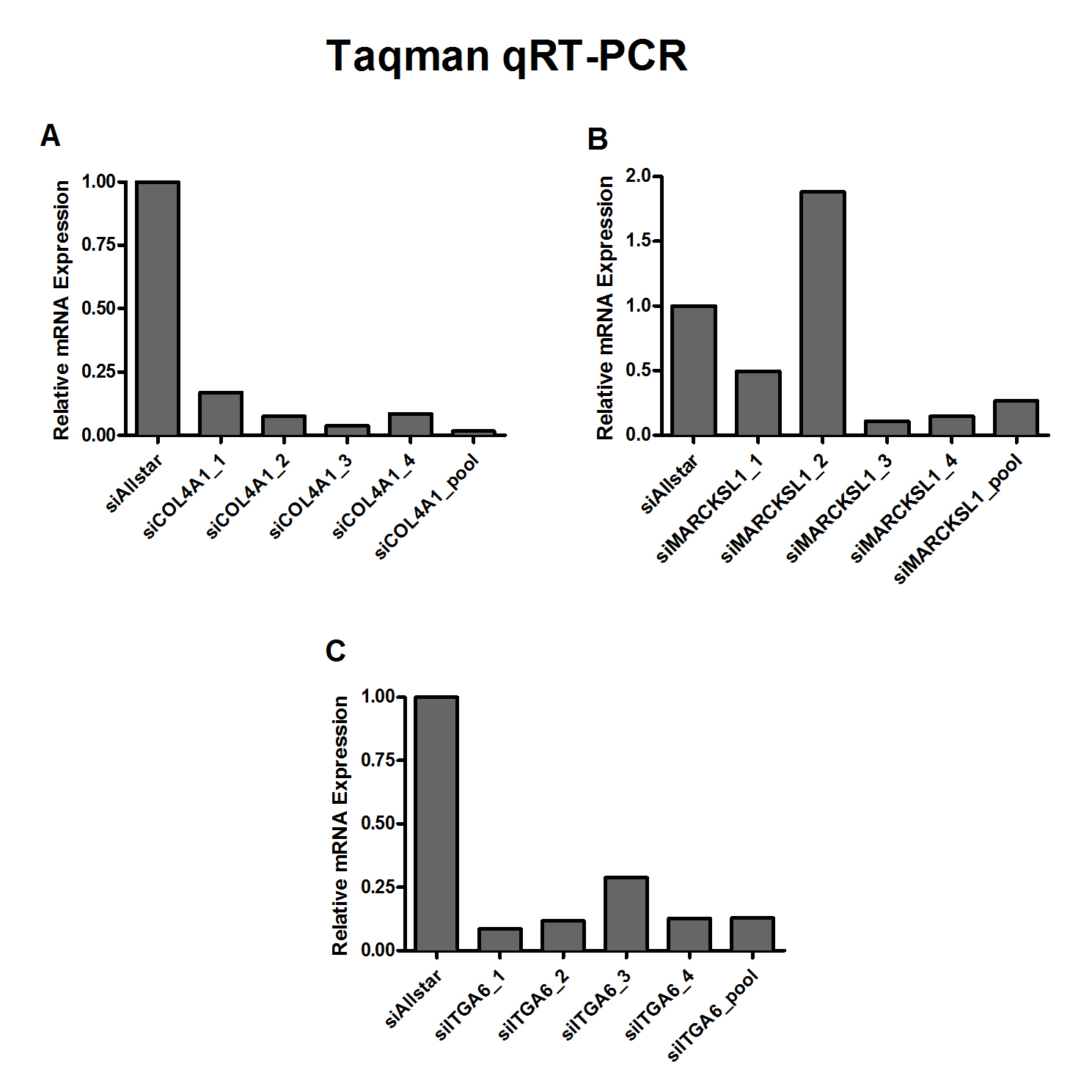

Supplement: Additional file 7: — Efficiency of target gene knockdown on the mRNA level using Taqman qRT-PCR. MCF10A cells were transfected with different siRNAs or siRNA negative control (siAllstar). 72 h later, cells were lysed and total mRNA was isolated and purified using RNeasy kit (Qiagen). The mRNA expression levels of the candidate genes were then assessed by Taqman qRT-PCR. Gene expression was normalized to HPRT and GAPDH housekeeping genes. Normalized gene expression is depicted as relative expression to cells transfected with siAllstar. Values represent the mean of three technical replicates. (JPG 350 kb) [file 12864_2016_2869_MOESM7_ESM.jpg]
