# Supplementary material for: The highly expressed 5’isomiR of hsa-miR-140-3p contributes to the tumor-suppressive effects of miR-140 by reducing breast cancer proliferation and migration
Source: BMC Genomics. 2016 Aug 8;17:566. doi: 10.1186/s12864-016-2869-x (PMC4977694; doi:10.1186/s12864-016-2869-x)
Supplement: Additional file 6: — List of primers and probes. This file contains all primer sequences used for cloning and mutagenesis of the luciferase reporter plasmids, as well as the UPL primers and probes used for qRT-PCR. (DOCX 17 kb) [file 12864_2016_2869_MOESM6_ESM.docx]

3’ UTR cloning primers

| Gene | Primer Orientation | Restriction Enzyme | Sequence |
| --- | --- | --- | --- |
| SRF | Forward | XhoI | ctcgagacctcccatttctgttgctg |
|  | Reverse | NotI | gcggccgctcccaggggaagtagaaacc |
| MARCKSL1 | Forward | XhoI | ctcgagggcaggtgggtgatctctaa |
|  | Reverse | NotI | gcggccgcccaggtggaagagaatggtt |
| CHCHD4 | Forward | XhoI | ctcgagttttggagtggaccttttgc |
|  | Reverse | NotI | gcggccgcacgtttcctctcttgctgct |
| COL4A1 | Forward | XhoI | ctcgagttgttaacagcaacgaacccta |
|  | Reverse | NotI | gcggccgctttaatgcgtctgtggcaat |
| CCT5 | Forward | XhoI | ctcgagaaatggatgtctcgtgatgc |
|  | Reverse | NotI | gcggccgccacagacccctcggataaga |
| DGCR8 | Forward | XhoI | ctcgagtctgaggagaccagcagtca |
|  | Reverse | NotI | gcggccgctgtgtgcacgattcataccc |
| CAP1 | Forward | XhoI | ctcgagaagtgccactgggttctttg |
|  | Reverse | NotI | gcggccgcaggcctcaggaaatgggtag |
| RUVBL1 | Forward | XhoI | ctcgagatggctgaggttttcagcag |
|  | Reverse | NotI | gcggccgcagagagagagagagagagaatcaaaa |
| ITGA6 | Forward | XhoI | ctcgagacccaaactgctttttccaa |
|  | Reverse | NotI | gcggccgcatcccattgctttggcacta |

Mutagenesis primers

| Gene | Primer Orientation | Sequence |
| --- | --- | --- |
| MARCKSL1 | Forward | cctcctctccctccacacgattctcccatcag |
|  | Reverse | ctgatgggagaatcgtgtggagggagaggagg |
| CHCHD4 | Forward | gtgtgtcctacccaaaccacacgccgccacttttgaattc |
|  | Reverse | gaattcaaaagtggcggcgtgtggtttgggtaggacacac |
| COL4A1 | Forward | gaaatcacaattttccacacgaataaagatggtcc |
|  | Reverse | Ggaccatctttattcgtgtggaaaattgtgatttc |
| DGCR8 | Forward | cacgctccttcccacacgccaacctgtggg |
|  | Reverse | cccacaggttggcgtgtgggaaggagcgtg |
| RUVBL1 | Forward | cctgggtccagccacacggcgcttgcccctg |
|  | Reverse | caggggcaagcgccgtgtggctggacccagg |
| ITGA6 | Forward | catgattagaaattaccacacgatatttgtataaaagtg |
|  | Reverse | cacttttatacaaatatcgtgtggtaatttctaatcatg |

Taqman primers and probes

| Gene | Primer Orientation | Sequence | Probe number |
| --- | --- | --- | --- |
| MARCKSL1 | Forward | ggcaggtgggtgatctctaa | 12 |
|  | Reverse | ccaggtggaagagaatggtt |  |
| CHCHD4 | Forward | ttttggagtggaccttttgc | 83 |
|  | Reverse | acgtttcctctcttgctgct |  |
| COL4A1 | Forward | ttgttaacagcaacgaacccta | 81 |
|  | Reverse | tttaatgcgtctgtggcaat |  |
| DGCR8 | Forward | aaaacttgcgaagaataaagctg | 47 |
|  | Reverse | tttaacaaagtcagggatgagga |  |
| ITGA6 | Forward | acccaaactgctttttccaa | 22 |
|  | Reverse | atcccattgctttggcacta |  |
